# MassARRAY-based simultaneous detection of hotspot somatic mutations and recurrent fusion genes in papillary thyroid carcinoma: the PTC-MA assay

**DOI:** 10.1007/s12020-017-1483-2

**Published:** 2017-12-06

**Authors:** Chiara Pesenti, Marina Muzza, Carla Colombo, Maria Carla Proverbio, Claudia Farè, Stefano Ferrero, Monica Miozzo, Laura Fugazzola, Silvia Tabano

**Affiliations:** 1 Division of Pathology, Fondazione IRCCS Ca’ Granda Ospedale Maggiore Policlinico, Milano, Italy; 2 Department of Pathophysiology and Transplantation, Università degli Studi di Milano, Milan, Italy; 3 Department of Biomedical, Surgical and Dental Sciences, Università degli Studi di Milano, Milan, Italy; 4 Division of Endocrine and Metabolic Diseases, IRCCS Istituto Auxologico Italiano, Milano, Italy

**Keywords:** Papillary thyroid carcinoma, MassARRAY, RET, NTRK1, BRAF, TERT

## Abstract

**Purpose:**

We exploited the MassARRAY (MA) genotyping platform to develop the “PTC-MA assay”, which allows the simultaneous detection of 13 hotspot mutations, in the *BRAF*, *KRAS*, *NRAS*, *HRAS*, *TERT*, *AKT1*, *PIK3CA*, and *EIF1AX* genes, and six recurrent genetic rearrangements, involving the *RET* and *TRK* genes in papillary thyroid cancer (PTC).

**Methods:**

The assay was developed using DNA and cDNA from 12 frozen and 11 formalin-fixed paraffin embedded samples from 23 PTC cases, together with positive and negative controls.

**Results:**

The PTC-MA assay displays high sensitivity towards point mutations and gene rearrangements, detecting their presence at frequencies as low as 5%. Moreover, this technique allows quantification of the mutated alleles identified at each tested locus.

**Conclusions:**

The PTC-MA assay is a novel MA test, which is able to detect fusion genes generated by genomic rearrangements concomitantly with the analysis of hotspot point mutations, thus allowing the evaluation of key diagnostic, prognostic, and therapeutic markers of PTC in a single experiment without any informatics analysis. As the assay is sensitive, robust, easily achievable, and affordable, it is suitable for the diagnostic practice. Finally, the PTC-MA assay can be easily implemented and updated by adding novel genetic markers, according to clinical requirements.

## Introduction

Papillary thyroid cancer (PTC) accounts for approximately 80% of thyroid cancers with an increasing prevalence over the last decade [1]. The extensive characterization of PTC reported in The Cancer Genome Atlas allowed the reclassification of PTC cases into molecular subtypes that better reflect their differential properties improving the pathological classification and clinical surveillance [2]. Given the established relevance of molecular characterization, clinical diagnosis is now facing the need for cost and time-effective techniques to investigate multiple molecular markers.

In the present study, we propose the mass spectrometry array (MassARRAY, MA) as a rapid, cost-effective, and sensitive method for the analysis of multiple hotspot point mutations concomitantly with fusion genes typical of PTC, in a single experiment. The procedure requires minimal amounts of material, representing an advantage for routine. Indeed, DNA fragments analyzed by MA are short, making it a useful technique for assessment of nucleic acid even from formalin-fixed paraffin-embedded (FFPE) samples. Furthermore, MA is a quantitative method, which provides data on the percentage of mutated allele and allows to identify mutations at low frequencies (5–10%) [3].

Exploiting the MA genotyping platform we designed an assay, the “PTC-MA assay”, to detect simultaneously 13 hotspot point mutations and six recurrent rearrangements, using both genomic DNA and cDNA from tumor samples. Detection of these fusion genes is possible because, although they derive from chromosome rearrangements that can involve different breakpoints at the DNA level, they generate identical fusion products at the transcription level.

## Materials and methods

### Samples

The following cases were evaluated:

- 23 non-consecutive PTCs harboring common genetic alterations (*BRAF*V600E, *TERT* G228A, *TERT* G250A, *NRAS* Q61R, *NRAS* Q61K, *HRAS* Q61K, *RET/PTC1*, *RET/PTC3*) recruited at Fondazione IRCCS Ca’ Granda (Table 1). All the alterations of the PTC samples were previously detected by Sanger sequencing, as already reported [4–6]. Samples were frozen tissues in 12 cases, and formalin-FFPE tissues in 11 cases. Five cases were analyzed starting from both frozen and FFPE samples (cases #1, 2, 5, 6 and 8). For frozen samples, tumors <1.5 cm were microdissected to ensure high tumor tissue content; in larger tumors, the core of the sample was macroscopically dissected. For FFPE samples, hematoxylin and eosin sections were evaluated to ensure a tumor cell content of at least 70%. To evaluate the accuracy of PTC-MA assay in quantifying mutated alleles, in samples #1 and #10, BRAF V600E mutation was assessed also by pyrosequencing (CE-IVD Anti-EGFR MoAb response BRAF status—Diatech Pharmacogenetics s.r.l., Jesi, Italy).

**Table 1:** Biological material analyzed by PTC-MA assay comprising 23 PTC samples, four cell lines and four lung tumor samples

ID/age at D	Biological Material	PTC Histologic variant	pTNM	Mutations							% of mutant alleles	Rearrangements	
				*BRAF*	*TERT*	*KRAS*	*HRAS*	*NRAS*	*AKT*	*PIK3CA*		*RET/PTC*	*N-TRK*
1/87	FS/FFPE	classical	pT3NXM0	***V600E***	WT	WT	WT	WT	WT	WT	**11** ^**a**^ **/8**	WT	WT
2/69	FS/FFPE	classical	pT3mN1bM0	***V600E***	***G228A***	WT	WT	WT	WT	WT	**30** **(** ***BRAF*** **)** ^**a**^ **/35 34** **(** ***TERT*** **)** ^**a**^ **/34**	WT	WT
3/63	FS	classical	pT3mN1aM0	WT	WT	WT	WT	WT	WT	WT		***RET/PTC1*** ^**a**^	WT
4/33	FS	classical	pT3mN1bM0	WT	WT	WT	WT	WT	WT	WT		***RET/PTC3*** ^**a**^	WT
5/52	FS/FFPE	follicular	pT3mNXM0	WT	WT	WT	WT	***Q61R***	WT	WT	**26** ^**a**^ **/25**	WT	WT
6/56	FS/FFPE	follicular	pT3mN1bM1	WT	***G250A***	WT	WT	WT	WT	WT	**24/22**	***RET/PTC3***	WT
7/61	FS	follicular	pT2N0M0	WT	WT	WT	***Q61K***	WT	WT	WT	**53**	WT	WT
8/60	FS/FFPE	classical	pT1N0M0	WT	WT	WT	WT	***Q61K***	WT	WT	**33** ^**a**^ **/35**	WT	WT
9/74	FS	classical	pT3mN1bM0	WT	***G228A***	WT	WT	WT	WT	WT	**47**	WT	WT
10/68	FS	Classical/follicular	pT4mN1bM0	***V600E***	***G228A***	WT	WT	WT	WT	WT	**21** **(** ***BRAF*** **)** ^**a**^ **24** **(** ***TERT*** **)** ^**a**^	WT	WT
11/41	FS	follicular	pT3N1bM0	WT	WT	WT	WT	WT	WT	WT	–	***RET/PTC1***	WT
12/22	FS	classical	pT1N1aM0	WT	WT	WT	WT	WT	WT	WT	–	***RET/PTC3***	WT
13/56	FFPE	classical	pT1N1aM0	WT	WT	WT	WT	***Q61R***	WT	WT	**20** ^**a**^	WT	WT
14/83	FFPE	classical	pT3mNXM0	WT	WT	WT	WT	***Q61K***	WT	WT	**28** ^**a**^	WT	WT
15/27	FFPE	classical	pT3mN1bM0	WT	WT	WT	***Q61R***	WT	WT	WT	**48**	WT	WT
16/62	FFPE	classical	pT3mN0M0	***V600E***	WT	WT	WT	WT	WT	WT	**27**	WT	WT
17/35	FFPE	classical	pT1mN1aM0	***V600E***	WT	WT	WT	WT	WT	WT	**19**	WT	WT
18 /28	FFPE	classical	pT2mN1aM0	***V600E***	WT	WT	WT	WT	WT	WT	**29**	WT	WT
19/50	FFPE	classical	pT3mN0M0	***V600E***	***G228A***	WT	WT	WT	WT	WT	**15** **(** ***BRAF*** **)** **23** **(** ***TERT*** **)**	WT	WT
20/41	FFPE	classical	pT1mNXM0	WT	***G228A***	WT	WT	WT	WT	WT	**37**	WT	WT
21/35	FFPE	classical	pT3mN1aM0	WT	***G228A***	WT	WT	WT	WT	WT	**29**	WT	WT
22/28	FFPE	follicular	pT2mNXM0	WT	WT	WT	WT	***Q61R***	WT	WT	**12**	WT	WT
23/35	FFPE	classical	pT2N1aM0	WT	WT	WT	WT	WT	WT	WT		***RET/PTC1***	WT
24	cell line	–	–	WT	WT	WT	WT	WT	WT	WT		WT	***TRK***
25	cell line	–	–	WT	WT	WT	WT	WT	WT	WT		WT	***TRK-T1***
26	cell line	–	–	WT	WT	WT	WT	WT	WT	WT		WT	***TRK-T3***
27	cell line	–	–	WT	WT	WT	WT	WT	WT	WT		***RET/PTC2***	WT
28	FFPE; C +	–	–	WT	WT	WT	WT	WT	***E17K***	WT	**46**	WT	WT
29	FFPE; C+	–	–	WT	WT	***G12V***	WT	WT	WT	WT	**48** ^**a**^	WT	WT
30	FFPE; C+	–	–	WT	WT	***G13C***	WT	WT	WT	WT	**54** ^**a**^	WT	WT
31	FFPE; C+	–	–	WT	WT	WT	WT	WT	WT	***E542K***	**72**	WT	WT

The percentages of the MA results are reported for point mutations

Genetic alterations and allelic frequencies are reported in bold.

*EIF1AX* was WT in all samples analyzed and no positive controls were available

*D* diagnosis, *FS* frozen sample, *FFPE* formalin fixed paraffin embedded sample *C+* positive control (lung tumor), *WT* wild-type

^a^ indicates samples analyzed in at least two independent experiments. The reported percentages of mutated allele are the average of the experiments (standard deviations are all above 8%)
- Three blood and 23 normal thyroid tissues contralateral to the tumor (12 frozen and 11 FFPE) served as negative controls.
- Positive controls, to validate detection of rare mutations not present in our PTC series: four FFPE lung cancer samples, two harboring mutations AKT1 E17K and PIK3CA E542K (cases #28 and #31, Table 1) previously detected by the CE-IVD MassArray Dx lung panel (Diatech Pharmacogenetics, Italy) and two with KRAS G12V and G13C (cases #29 and #30, Table 1) previously assessed by pyrosequencing with the CE-IVD Anti-EGFR MoAb response KRAS status (Diatech Pharmacogenetics, Italy). Unfortunately, no positive controls were available for the EIF1AX mutation.
- Commercial DNA reference standards HORIZON (Cambridge, UK), harboring the BRAF V600E or the NRAS Q61R mutations at known percentages (10 and 5%), to study the sensitivity of the PTC-MA assay in detecting point mutations.
- Positive controls to validate detection of fusion genes not present in the PTC series: four mouse NIH 3T3 fibroblasts cell lines, transfected with human DNA harboring the fusion genes, *TRK*, *TRK-T1*, *TRK-T3*, and *RET/PTC2* [7–10]. These positive controls were also exploited to test the sensitivity of the PTC-MA assay in detecting fusion genes.

The study was approved by the Ethical Committee of the Institution involved.

### DNA/RNA extraction, and reverse transcription

Genomic DNA was extracted from either frozen, FFPE tissues (Puregene® Core Kit A, Qiagen, Germantown, MD, USA), peripheral blood samples (Illustra Nucleon Bacc2, GE Healthcare, Barrington, IL, USA).

Total RNA was extracted from frozen or FFPE tissue samples using a Trizol-based commercial kit (Thermo Fisher, Waltham, MA, USA), and from peripheral leukocytes by the PAX gene Blood RNA System (PreAnalytiX, Hombrechtikon, Switzerland). One microgram of each RNA sample was reverse-transcribed using a Superscript reverse transcriptase II Kit (Thermo Fisher), with a hexamer mixture as primers. β-actin amplification from the cDNA was performed as an internal quality control. The optimal amount of DNA and cDNA for MA analysis is 30 ng; however, when the genetic material is limited, the system can work with 10 ng of starting material, as in the manufacturer’s protocol [11].

DNA and cDNA of cell lines were provided by Istituto Nazionale Tumori of Milan.

### PTC-MA assay

Thirteen hotspot mutations and the six recurrent fusion genes, representing the most frequently observed in PTC [2], were included in the PTC-MA assay (Supplemental Table 1).

Three multiplex polymerase chain reaction (PCR) reactions were designed: Mix 1 was performed using DNA and with intronic/exonic PCR primers for point mutations identification, since the primers for *TERT*, *EIF1AX* and *AKT1* were located in non-coding sequences. Mixes 2 and 3 were used to analyze cDNA, with exonic primers for the identification of both point mutations located within exons and fusion genes. For fusion genes, PCR primers were designed in order to obtain a product only in the presence of the rearrangement. Extension primers spanned the fusion gene transcripts and included parts of both genes. With the aim to minimize the number of reaction mix needed to complete the PTC-MA assay, in order to reduce costs and time of execution, some single nucleotide variant (4 out of 9) were analyzed together with some fusions starting from cDNA. The multiplexing scheme, the PCR conditions and the primers sequences are listed in Supplemental Table 1.

DNA/cDNA were used for PCR, SAP (shrimp alkaline phosphatase), and single-base extension (SBE) reactions; SBE products were analyzed using MassARRAY Typer 4.0 software (all Agena Bioscience, San Diego, CA, USA), following manufacturer’s protocol.

## Results

The effectiveness and specificity of the multiplex reactions were firstly determined analyzing all mutations and rearrangements on negative control samples (23 normal thyroid tissue samples, and 3 blood samples). As expected, all controls were wild-type and no PCR/extension products were obtained using the fusion gene-specific primers, indicating the absence of rearrangements (data not shown).

To optimize the multiplex PCR panel, primers were designed to detect 4 out of 9 exonic point mutations on cDNA (Supplemental Table 1). This was possible, since we previously verified that in two PTC samples (cases #2 and 5), harboring BRAF V600E and NRAS Q61R respectively, the percentage of mutated alleles found in DNA or cDNA were similar (Supplemental Fig. 1), demonstrating that the reaction template (DNA or cDNA) did not influence the point mutations detection.

To test possible differences in the results due to sample type (frozen or FFPE), the PTC-MA assay was performed in five cases (#1, 2, 5, 6 and 8) in both frozen and FFPE samples, giving superimposable percentages of mutated alleles, thus indicating that PTC-MA assay is highly reliable also on archival material (Table 1).

**Fig. 1:**
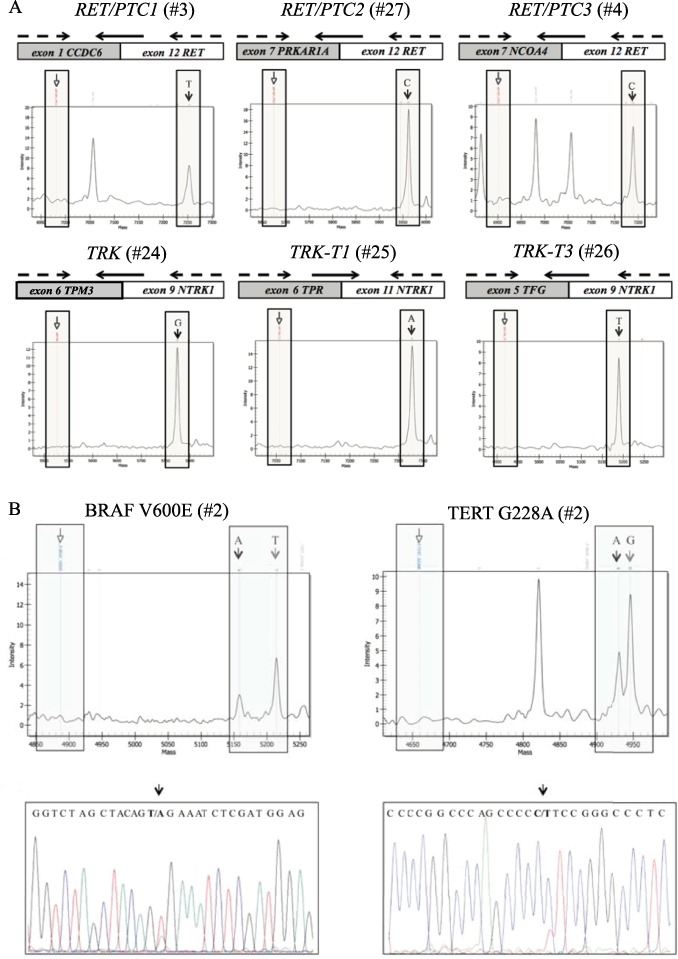
MA spectra for gene rearrangements and hotspot mutations. **a** Schematic representation of PCR and extension primers for the detection of fusion genes and representative MA spectra. The chromosomal regions involved in the formation of fusion genes are indicated in white (*RET* and *NTRK1*) and gray (*CCDC6*, *NCOA4*, *TPM3*, *TPR*, *PRKAR1A*, and *TFG*), and are reported on the top of the corresponding MA spectrum. The positions of the PCR and extension primers are indicated by dotted and black arrows, respectively. In each spectrum, the empty arrow indicates the position of the unextended primer (when the fusion gene is absent); the black arrow points to the position of the extension product, in presence of the fusion gene. The spectra of *RET/PTC1* and *RET/PTC3* were generated from the positive tissue samples, (#3 and #4, Table 1), and the spectra of *RET/PTC2*, *TRK*, *TRK-T1* and *TRK-T3* were generated from corresponding positive NIH 3T3 cell lines (#24–27, Table 1). **b** MA spectra of the coexistent *BRAF* V600E and *TERT* G228A mutations and the corresponding Sanger sequences in sample #2. On the top, the MA spectra for both *BRAF* V600E and *TERT* G228A. In each spectrum, the black arrow indicates the mutated allele, the gray arrow the wild-type allele, and the empty arrow indicates the position of the extension primer. The corresponding electropherograms obtained by Sanger sequencing are reported below. Black arrows point to the mutated bases. For *TERT* assays, MA and Sanger sequencing were designed to assess the reverse and forward strands of the gene, respectively

The PTC-MA assay was then used to test 23 PTC samples harboring diverse genetic alterations and positive controls. As detailed in Table 1, all the point mutations and gene rearrangements were detected by the PTC-MA assay. The main advantage of this assay is the possibility to test recurrent gene rearrangements with always same fusion products, as those typical of PTC. Notably, all *RET* and *NTRK1* rearrangements included in the assay were clearly identified (Fig. 1a).

As shown in Fig. 1b, the co-occurrence of two genetic alterations was also clearly detected by the PTC-MA assay, as for cases #2, 10 and 19 (harboring both *BRAF* V600E and *TERT* G228A mutations) and for case #6 (*RET/PTC3* rearrangement and *TERT* G250A mutation).

In addition, for BRAF V600E (cases #1 and 10) and KRAS mutations (cases #29 and 30), we found similar percentages of the mutated alleles comparing PTC-MA assay and CE-IVD kits based on pyrosequencing, which is considered a gold-standard method for allelic quantification (Supplemental Table 2).

Finally, the sensitivity of the assay in detecting point mutations was verified by commercial DNA reference standards HORIZON for BRAF V600E and NRAS Q61R (harboring known rates, 10 and 5%, of mutated allele). As expected [3], the PTC-MA assay was able to detect even 5% of mutated alleles (Supplemental Fig. 2a). Moreover, we assessed the sensitivity for the detection of fusion genes using the cells lines harboring *RET* and *TRK* rearrangements (cases #24–27; Table 1). By serial dilutions with the tumor sample 28, which is negative for *RET* and *TRK* rearrangements, we demonstrated that the PTC-MA assay is able to detect the presence of fusion genes with high sensitivity, also when they were present in 5% only of the total cDNA (Supplemental Fig. 2b).

## Discussion

Reliable, robust and affordable technology for genetic characterization of cancers is important in supporting routine clinical practice. The MA technology has recently been approved for clinical diagnosis, allowing the identification of mutations with high sensitivity [3, 12, 13].

Here, we report a novel MA assay, the PTC-MA assay, able to detect 13 point mutations and six gene rearrangements typical of PTC in a single experiment, thereby saving time (2 working-days) and reducing costs (approximately 60 euros per sample). It is worth to note that this PTC-MA assay was developed neither to search for novel genetic alterations in PTC, nor to test for all known genetic alterations, but to detect the point mutations and gene fusions most frequently involved in PTC carcinogenesis. Focusing on the clinical utility of the genetic analysis in PTC (more accurate diagnosis, selection of targeted treatments), we believe that NGS technologies or the wide panels of analysis can add little information to our PTC-MA assay, in spite of significantly higher costs comparing with MA [14]. Moreover, NGS is not affordable for small/medium-sized laboratories, and is thus only rarely applied. Conversely, our PTC-MA assay could be exploited and developed by several laboratories, leading to a better characterization of PTCs, with a positive clinical and therapeutic impact.

PTC-MA assay is appropriate for routine diagnosis because: (i) it is sensitive also for sample with few cancer cells; (ii) it is accurate in determining the percentages of mutated alleles; (iii) it can be easily implemented and updated by adding novel genetic markers, according to clinical requirements.

In conclusion, though these results will be further validated in a larger cohort of cases, the PTC-MA assay is useful and robust for the simultaneous detection of hotspot point mutations and rearrangements in PTC, using DNA and RNA isolated from frozen as well as FFPE samples.

## Electronic supplementary material

Supplemental Figure 1

Supplemental Figure 2

Supplemental Table 1

Supplemental Table 2
