# Supplementary figures and images for: MassARRAY-based simultaneous detection of hotspot somatic mutations and recurrent fusion genes in papillary thyroid carcinoma: the PTC-MA assay
Source: Endocrine. 2017 Dec 6;61(1):36–41. doi: 10.1007/s12020-017-1483-2 (PMC5997117; doi:10.1007/s12020-017-1483-2)

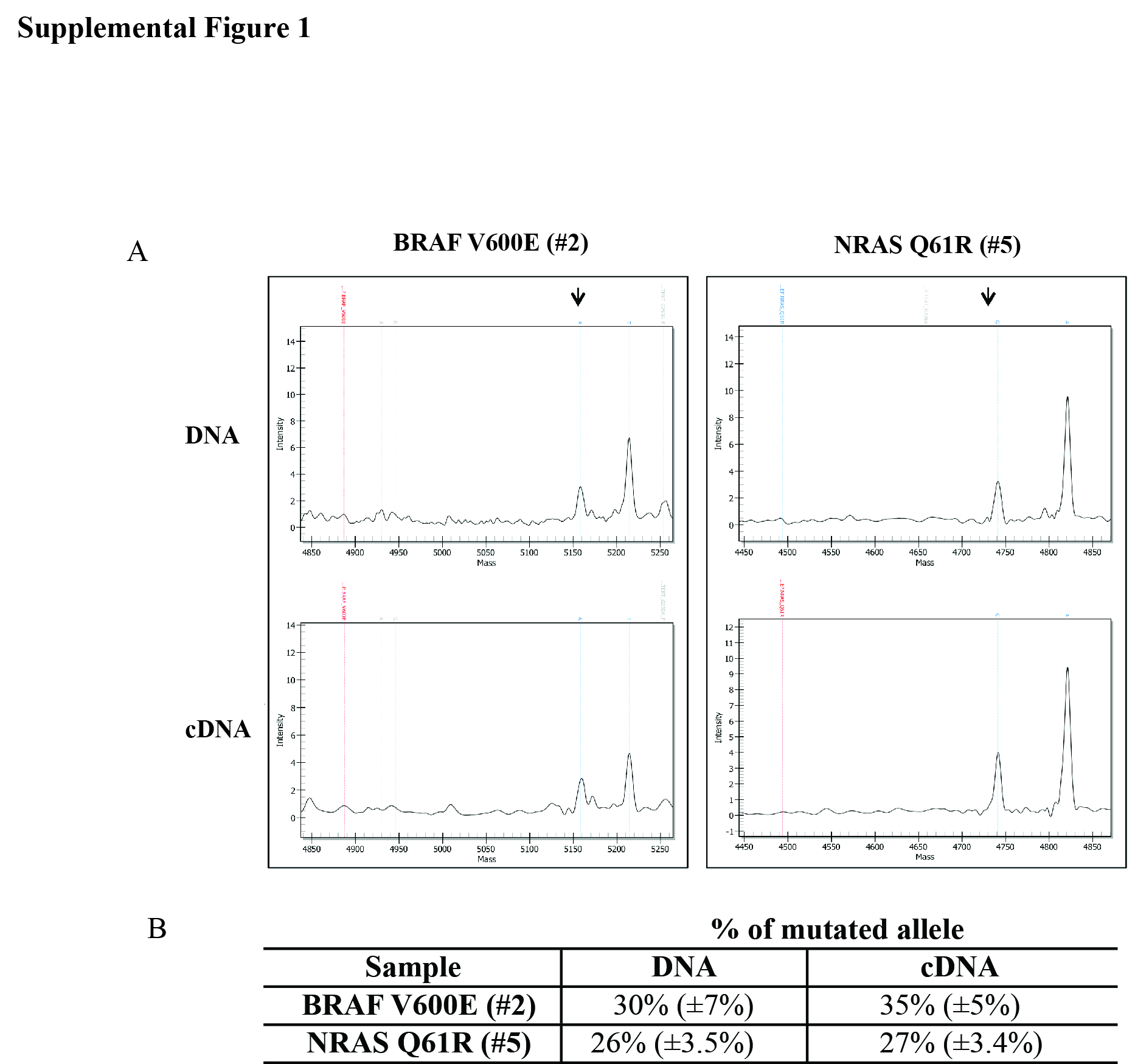

Supplement: Supplementary file 1 — Supplemental Figure 1 [file 12020_2017_1483_MOESM1_ESM.tif]

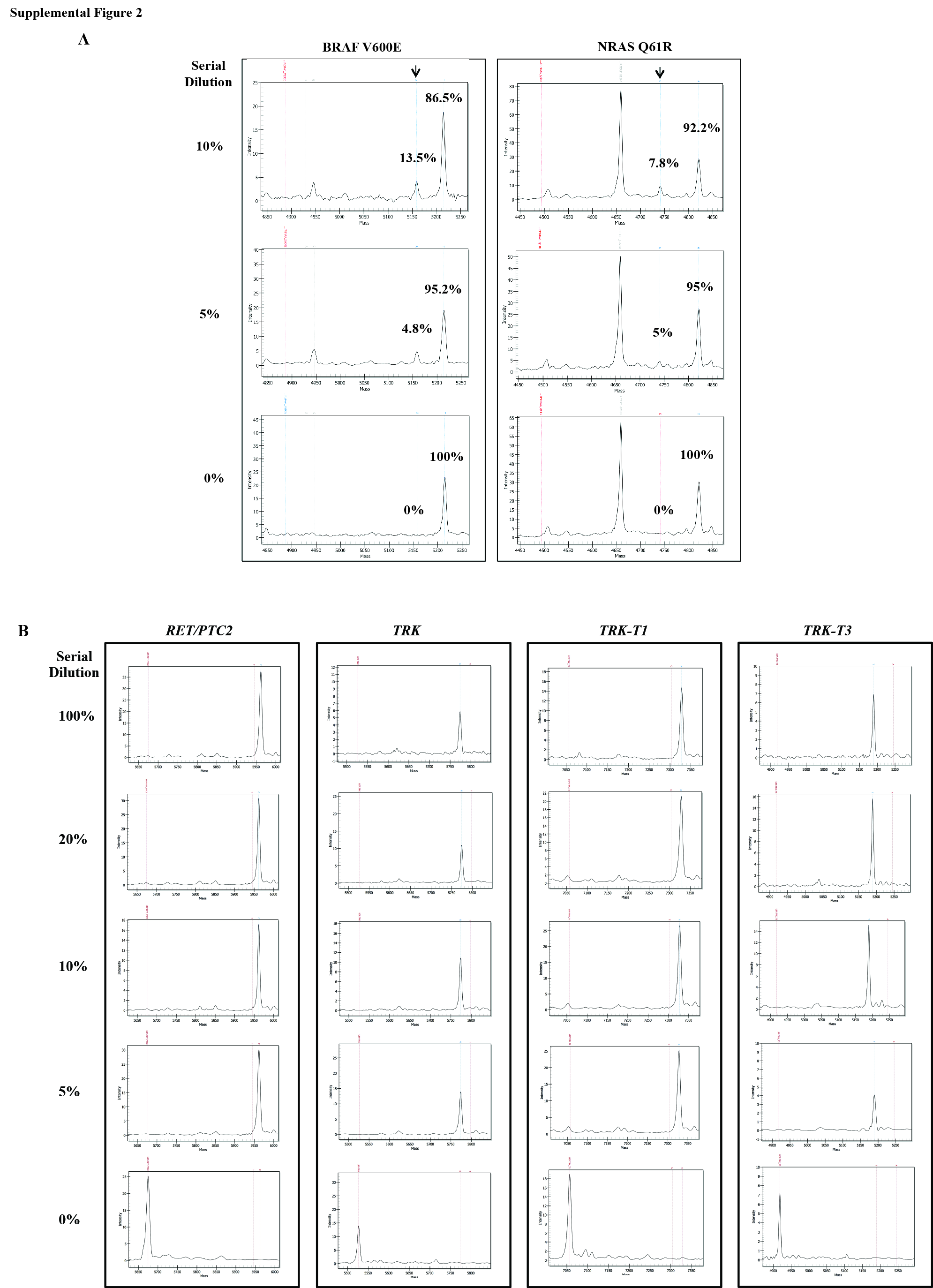

Supplement: Supplementary file 2 — Supplemental Figure 2 [file 12020_2017_1483_MOESM2_ESM.tif]
