# Supplementary material for: MassARRAY-based simultaneous detection of hotspot somatic mutations and recurrent fusion genes in papillary thyroid carcinoma: the PTC-MA assay
Source: Endocrine. 2017 Dec 6;61(1):36–41. doi: 10.1007/s12020-017-1483-2 (PMC5997117; doi:10.1007/s12020-017-1483-2)
Supplement: Supplementary file 3 — Supplemental Table 1 [file 12020_2017_1483_MOESM3_ESM.docx]

**Supplemental Table 1 -** Sequences of custom-designed primers for mass spectrometry analysis of genomic mutations/gene rearrangements.

| ***Sample analyzed*** | ***Multiplexed PCR***  ***(Mix ID)*** | ***Mutation/ rearrangement detectable*** | ***RefSeq^#^*** | ***PCR forward primer (5′→3′)****** | ***PCR reverse primer (5′→3′)****** | ***Amplicon size (bp)*** | ***Extension***  ***primer (5′→3′)*** | ***Extension primer direction*** |
| --- | --- | --- | --- | --- | --- | --- | --- | --- |
| Genomic DNA | 1 | BRAF_V600E | NM_004333 | ACGTTGGATG  TATATTTCTT  CATGAAGACC | ACGTTGGATG  TTCAAACTGA  TGGGACCCAC | 95 | TTTGGTCTAG  CTACAG | F |
|  | 1 | AKT1_E17K | NM_005163 | ACGTTGGATG  TCTGACGGGT  AGAGTGT | ACGTTGGATG  TCATTCTTGA  GGAGGAAGT | 96 | GTGGCCGCCA  GGTCTTGATG  TACT | R |
|  | 1 | EIF1AX_c338-1GtoC | NM_001412 | ACGTTGGATG  TTACAGTGCT  GACTTATGAG | ACGTTGGATG  TCTCCAGGAC  CAAATGTATC | 98 | AGTATTTCTT  ACTGTGTCCT  TA | F |
|  | 1 | NRAS_Q61R | NM_002524 | ACGTTGGATG  CCTGTCCTCA  TGTATTGGTC | ACGTTGGATG  GTGAAACCTG  TTTGTTGGAC | 82 | GCACTGTACT  CTTCT | R |
|  | 1 | NRAS_Q61K | NM_002524 |  |  |  | GACATACTGG  ATACAGCTGGA | F |
|  | 1 | HRAS_Q61K | NM_176795 | ACGTTGGATG  CAAACACACA  CAGGAAGCCC | ACGTTGGATG  CTGTTGGACA  TCCTGGATAC | 113 | ATCCTGGATA  CCGCCGGC | F |
|  | 1 | HRAS_Q61R | NM_176795 |  |  |  | GCATGGCGCT  GTACTCCTCC | R |
|  | 1 | TERT_G228A | NM_001193376 | ACGTTGGATG  GAAAGGAAGG  GGAGGGGCT | ACGTTGGATG  TTCACCTTCC  AGCTCCGCCT | 108 | CTGGGAGGGC  CCGGA | F |
|  | 1 | TERT_G250A | NM_001193376 |  |  |  | CTGGGCCGGG  GACCCGG | F |
| cDNA | 2 | HRAS_G13C | NM_176795 | ACGTTGGATG  GACGGAATAT  AAGCTGGTGG | ACGTTGGATG  GTTCTGGATC  AGCTGGATGG | 76 | GTCAGCGCAC  TCTTGCCCACAC | R |
|  | 2 | KRAS_G12V | NM_004985 | ACGTTGGATG  AGGCCTGCTG  AAAATGACTGA | ACGTTGGATG  GCTGTATCGT  CAAGGCACTC | 80 | TTGTGGTAGT  TGGAGCTG | F |
|  | 2 | RET_PTC1 | NM_005436 + NM_020975 | ACGTTGGATG  AACCAAGTTC  TTCCGAGGGA | ACGTTGGATG  ACCTACAAACT  GAAGTGCAAGG | 108 | TCCCACTTTG  GATCCTCGAT  GGT | R |
|  | 2 | RET_PTC3 | NM_001145261 + NM_020975 | ACGTTGGATG  AGGACTGGCT  TACCCAAAAG | ACGTTGGATG  AACCAAGTTC  TTCCGAGGGA | 80 | TCCCACTTTG  GATCCTCCTG  ACT | R |
|  | 2 | TRK | NM_001278190 + NM_001012331 | ACGTTGGATG  AGTTTGCTGA  GAGATCGGT | ACGTTGGATG  TGTTTCGTCC  TTCTTCTCCA | 101 | ATGTGCTGTT  AGTGTCTT | R |
|  | 2 | TRK_T1 | NM_003292 + NM_001012331 | ACGTTGGATG  ACGCTTGGAG  CAAGAAAAGG | ACGTTGGATG  GCATCACTGA  AGTATTGTGG | 79 | AAAGGAATTG  CTACATAGGC  TCC | F |
| cDNA | 3 | PIK3CA_E542K | NM_006218 | ACGTTGGATG  TTTCTACACG  AGATCCTCT | ACGTTGGATG  TGTGACTCCA  TAGAAAATC | 63 | CCTGCTCAGT  GATTT | R |
|  | 3 | RET_PTC2 | NM_002734 + NM_020975 | ACGTTGGATG  ACAAATGTGAAATTGTGGGGCA | ACGTTGGATG  AACCAAGTTC  TTCCGAGGGA | 93 | CCCACTTTGG  ATCCTCCAT | R |
|  | 3 | KRAS_G13C | NM_004985 | ACGTTGGATG  AGGCCTGCTG  AAAATGACTGA | ACGTTGGATG  GCTGTATCGT  CAAGGCACTC | 80 | AAGGCACTCT  TGCCTACGC | R |
|  | 3 | TRK_T3 | NM_001195478 + NM_001012331 | ACGTTGGATG  CAGCAAGTAT  GTCTGCTTTT  GATCCT | ACGTTGGATG  TGTTTCGTCC  TTCTTCTCCA | 143 | TGCTGTTAGT  GTCTGA | R |

***^#^*** From Ensembl database (http://www.ensembl.org), genome assembly GRCh38p.q10 (accessed, May 2017); *All PCR primers have a 10mer tag (ACGTTGGATG-) at the 5' end, as specified in the Agena Bioscience (Hamburg, Germany) protocol.
