# Supplementary material for: MassARRAY-based simultaneous detection of hotspot somatic mutations and recurrent fusion genes in papillary thyroid carcinoma: the PTC-MA assay
Source: Endocrine. 2017 Dec 6;61(1):36–41. doi: 10.1007/s12020-017-1483-2 (PMC5997117; doi:10.1007/s12020-017-1483-2)
Supplement: Supplementary file 4 — Supplemental Table 2 [file 12020_2017_1483_MOESM4_ESM.docx]

**Supplemental Table 2: Comparison between the percentages of mutated alleles detected by the PTC-MA assay and pyrosequencing.**

|  |  | **% of mutated allele** | |
| --- | --- | --- | --- |
|  | **Sample** | **PTC-MA assay** | **Pyrosequencing** |
| **BRAF V600E** | **1** | 11% (±1.4%) | 11% (±0.1%) |
|  | **10** | 21% (±3.9%) | 17% (±0.7%) |
| **KRAS G12V** | **29** | 52% | 45% (±5%) |
| **KRAS G13C** | **30** | 54% (±7.7%) | 45% (±8%) |

The results of at least two independent experiments are reported as mean ± standard deviations.
